# Unusual presentation of a patient with complement deficiency and immunoglobulin deficiency

**DOI:** 10.1186/1710-1492-10-S1-A39

**Published:** 2014-03-03

**Authors:** Vaishaali Manga, Donald Stark

**Affiliations:** 1 Department of Internal Medicine, Memorial University, St. John’s, Newfoundland and Labrador, A1B3V6, Canada; 2 Department of Clinical Immunology and Allergy, University of British Columbia, Vancouver, British Columbia, V6Z1Y6, Canada

## Background

The complement system is a vital component of innate immunity. Deficiencies in any part of the complement pathway characteristically present with recurrent infections. C2 factor deficiency is the most common complement deficiency. The presentation can vary from being asymptomatic to developing recurrent infections. Thus empiric vaccinations have been recommended, despite the lack of substantial evidence to support this practice. Recently, Jonnson and colleagues revisited this controversial topic and demonstrated the importance of vaccinations in this patient population. They further established that C2 deficient patients can mount an immune response to vaccination, undergo class-switching and develop a ‘more efficient’ phagocytosis.^1^

C2 deficiency is inherited in an autosomal recessive pattern. Homozygotes generally present with increased severity of disease as compared to heterozygotes. Alper and colleagues state that 25% of C2-deficient homozygotes have increased susceptibility to severe bacterial infections.^2^ Furthermore, they found that these C2-deficient patients had significantly lower mean levels of IgG4 and IgA than those patients that did not demonstrate an increased susceptibility for recurrent infections.

## Case

We report a case of a 59-year-old female with a history of C2 deficiency presenting with recurrent upper and lower respiratory tract infections. Although she received the pneumococcal vaccine, she failed to mount a response based on her post-vaccination titers. Further workup revealed a borderline low IgG. Due to her having recurrent infections and a borderline low IgG, the patient was started on subcutaneous immunoglobulin (SCIG) therapy as a trial. While on the treatment, she did not develop any new infections.

## Discussion

In the cohort by Jonnson, post-vaccination C2 deficient patients mounted a good response to vaccination along with a ‘more efficient’ phagocytosis via increased opsonin production secondary to a possible c1q dependent C2-independent pathway [1]. This pathway may not be effectively activated in post vaccinated C2 patients who are IgG deficient because of the role IgG plays in the c1q dependent pathway. Therefore, C2 deficient patients that have a concomitant immunoglobulin deficiency may respond differently to vaccination than the C2 deficient patient with normal immunoglobulins. We postulate that our patient did not respond to antigenic stimulations because she may fall in the 25% C2 deficient homozygous category in which the IgG levels are decreased [2].

## Conclusion

Further studies are requires to determine the effectiveness of vaccination in these two different C2 deficient patient populations and to help guide vaccination protocols.
